# Diversity and Postzygotic Evolution of the Mitochondrial Genome in Hybrids of *Saccharomyces* Species Isolated by Double Sterility Barrier

**DOI:** 10.3389/fmicb.2020.00838

**Published:** 2020-05-07

**Authors:** Adrienn Szabó, Zsuzsa Antunovics, Edina Karanyicz, Matthias Sipiczki

**Affiliations:** Department of Genetics and Applied Microbiology, University of Debrecen, Debrecen, Hungary

**Keywords:** *Saccharomyces*, hybrid, sterile, mitotype, fermentation, respiration, alloploid, meiosis

## Abstract

Eukaryotic species are reproductively isolated by sterility barriers that prevent interspecies fertilization (prezygotic sterility barrier) or the fertilization results in infertile offspring (postzygotic sterility barrier). The *Saccharomyces* species are isolated by postzygotic sterility barriers. Their allodiploid hybrids form no viable gametes (ascospores) and the viable ascospores of the allotetraploids cannot fertilize (conjugate). Our previous work revealed that this mechanism of reproductive isolation differs from those operating in plants and animals and we designated it double sterility barrier (the failure of homeologous chromosomes to pair and the repression of mating by mating-type heterozygosity). Other studies implicated nucleo-mitochondrial incompatibilities in the sterility of the *Saccharomyces* hybrids, a mechanism assumed to play a central role in the reproductive isolation of animal species. In this project the mitochondrial genomes of 50 cevarum (*S. cerevisiae* × *S. uvarum*) hybrids were analyzed. 62% had *S. cerevisiae* mitotypes, 4% had *S. uvarum* mitotypes, and 34% had recombinant mitotypes. All but one hybrid formed viable spores indicating that they had genomes larger than allodiploid. Most of these spores were sterile (no sporulation in the clone of vegetative descendants; a feature characteristic of allodiploids). But regardless of their mitotypes, most hybrids could also form fertile alloaneuploid spore clones at low frequencies upon the loss of the *MAT*-carrying chromosome of the *S. uvarum* subgenome during meiosis. Hence, the cevarum alloploid nuclear genome is compatible with both parental mitochondrial genomes as well as with their recombinants, and the sterility of the hybrids is maintained by the double sterility barrier (determined in the nuclear genome) rather than by nucleo-mitochondrial incompatibilities. During allotetraploid sporulation both the nuclear and the mitochondrial genomes of the hybrids could segregate but no correlation was observed between the sterility or the fertility of the spore clones and their mitotypes. Nucleo-mitochondrial incompatibility was manifested as respiration deficiency in certain meiotic segregants. As respiration is required for meiosis-sporulation but not for fertilization (conjugation), these segregants were deficient only in sporulation. Thus, the nucleo-mitochondrial incompatibility affects the sexual processes only indirectly through the inactivation of respiration and causes only partial sterility in certain segregant spore clones.

## Introduction

The biological species are defined as groups of interbreeding natural populations that are reproductively isolated from other such groups (Mayr, 1942). Because of the reproductive isolation, interspecies hybridisation is a rare event in most groups of eukaryotes and usually results in infertile offspring. Under this concept, two interbreeding taxa (populations) are conspecific, if they produce fertile hybrids and allospecific, if they fail to produce zygotes (prezygotic sterility barrier) or their hybrids are infertile (postzygotic sterility barrier). Interspecies hybrids occur quite commonly in closely related, vegetatively propagating groups of species such as certain yeasts and fungi (for clonal propagation, gametes are not needed) or tolerate high ploidy (e.g., certain groups of higher plants; allopolyploid plants can produce viable diploid gametes). It is assumed that most extant plant species have emerged via interspecies hybridisation (Soltis et al., 2009).

The budding yeast genus *Saccharomyces* evolved from an ancient whole genome duplication event (Marcet-Houben and Gabaldon, 2015; Wolfe, 2015) is an excellent model system for the investigation of interspecies hybridisation. It accommodates seven “natural” species and two highly heterogeneous “hybrid” species of diverse chimeric genomes (e.g., Naseeb et al., 2017 and references therein). The species of the genus are reproductively isolated by a postzygotic double sterility barrier (Pfliegler et al., 2012). Under laboratory conditions any of the *Saccharomyces* species can form hybrids with any other species of the genus (e.g., Banno and Kaneko, 1989; Hawthorne and Philippsen, 1994; Naumov, 1996; Marinoni et al., 1999; Lorenz et al., 2002; Antunovics et al., 2005; Solieri et al., 2005; Rainieri et al., 2008; Bellon et al., 2011, 2013; Garcia Sanchez et al., 2012; Pérez-Través et al., 2014; Lopandic et al., 2016; Karanyicz et al., 2017; Nikulin et al., 2018; Peris et al., 2019; Sipiczki, 2019; Lairón-Peris et al., 2020). The interspecies hybrids are viable but sterile because cannot produce functional gametes (ascospores). The allodiploid two-species hybrids having single sets of parental chromosomes either do not produce ascospores or if they do so, the spores are very rarely viable (e.g., Hawthorne and Philippsen, 1994; Kishimoto, 1994; Hunter et al., 1996; Zambonelli et al., 1997; Marinoni et al., 1999; Liti et al., 2006; Kunicka-Styczyńska and Rajkowska, 2011; Pfliegler et al., 2012).

According to the most widely accepted model, the sporulation deficiency of the *Saccharomyces* allodiploid hybrids is due to the inability of the chromosomes of the subgenomes to pair correctly in prophase-I of meiosis. The failure of the allosyndetic (homeologous) chromosomes to pair is the first part of the double sterility barrier (for a review, see Sipiczki, 2018). It can be overcome by genome duplication. Much like in the case of the hybrids of many plant species, the duplication of the *Saccharomyces* hybrid genome “normalizes” meiosis because each chromosome will have a homologous (autosyndetic) partner to pair with. The allotetraploid meiosis produces viable allodiploid spores which, however, are sterile as the allodiploid hybrids. The sterility of the cells produced by these spores (F1 spore clones) is the second part of the double sterility barrier and is due to *MAT* heterozygosity (Pfliegler et al., 2012) that suppresses the activity of the genes required for mating (fertilization) (Herskowitz, 1988). Thus, genome duplication does not turn the interspecies hybrid fertile. It only provides a possibility for genomic changes that result in fertile alloaneuploid and chimeric derivatives by a process called GARMe (Genome Autoreduction in Meiosis) (Karanyicz et al., 2017). The key event that launches GARMe is the loss of the *MATa/MATalpha* heterozygosity that unblocks the processes which are required for the cells to function as gametes (an important difference from the allodiploid products of the plant allotetraploid meiosis). The *MAT* heterozygosity is lost when the meiotic division machinery fails to transfer copies of the *MAT*-carrying chromosomes of both subgenomes to a spore. The resulting alloaneuploid spore nullisomic for one parental *MAT*-carrying chromosome forms a clone of fertile vegetative cells with reactivated mating-type switching and mating programs. These cells can mate with each other within the clone and the resulting “intraclonal hybrids” form fertile F2 spore clones. The intraclonal mating and sporulation of the F2 spore clone then will give rise to additional fertile filial generations. Meanwhile additional chromosomes can be lost and recombination can also take place. Thus, GARMe produces diverse chimeric genomes similar to those found in “natural hybrids” of wine yeasts (for a review, see Sipiczki, 2018).

Less is known about the fate of the mitochondrial genome during the process of interspecies hybridisation. Most of the de-novo hybridisation studies found the synthetic hybrids homoplasmic. The hybrid cells received mtDNA from only one of the parental species and the donor was usually the same in all hybrids produced by the same combination of parental strains (e.g., Marinoni et al., 1999; Pulvirenti et al., 2000; De Vero et al., 2003; Antunovics et al., 2005; Lee et al., 2008; Rainieri et al., 2008; Solieri et al., 2008; Garcia Sanchez et al., 2012; Albertin et al., 2013; Karanyicz et al., 2017; Li and Fay, 2017; Origone et al., 2018; Verspohl et al., 2018). For example, in *Saccharomyces cerevisiae x S. uvarum* hybridisation, the “cevarum” hybrids had *S. cerevisiae* mitotypes (e.g., Antunovics et al., 2005), whereas in the *S. kudriavzevii x S. uvarum* combination the resulting “kudvarum” hybrids had *S. uvarum* mtDNA (Karanyicz et al., 2017). In the synthetic interspecies hybrids an interesting correlation was noticed between the origin of the mitochondrial genome and the higher stability of one of the nuclear subgenomes in GARMe. In cevarum hybrids having *S. cerevisiae* mitotypes, the *S. cerevisiae* subgenome was more stable (Antunovics et al., 2005), in kudvarum hybrids having *S. uvarum* mitotypes, the *S. uvarum* subgenome was more stable (Karanyicz et al., 2017). Consistent with these findings, a recent study involving six species also found that in the instable hybrids the mitochondrial genome donor tended to retain more nuclear chromosomes (Peris et al., 2019). Similar trends can also be observed in the nature. Recent studies analyzed the mitochondrial genomes of large numbers of chimeric (hybrid) brewing yeasts and also found correlation between mitotypes and the structure of the nuclear genomes, but did not address the impact of the mitotypes on the sexual activities (reproductive barrier) of the strains (Langdon et al., 2019). These observations hint at the possibility that the mitotype of the hybrid may affect the direction of the postzygotic evolution of the hybrid nuclear genome perhaps by impairing the fitness and viability of spores inheriting allospecific (mismatching) combinations of alleles of certain nuclear and mitochondrial genes. This notion fits well with the incompatibilities discovered between certain chromosomal and mitochondrial genes of *S. cerevisiae* and *S. uvarum*. For example in a cevarum hybrid having *S. cerevisiae* mitotype, the lack of the *S. cerevisiae AEP2* gene in the nucleus was found to cause respiration deficiency because its *S. uvarum* ortholog is incompatible with the *OLI1*/*ATP9* gene of the *S. cerevisiae* mitochondrion (Lee et al., 2008). The *S. uvarum* Aep2 protein cannot regulate the translation of the *S. cerevisiae ATP9* mRNA. Similar incompatibility was detected between the S. *uvarum* nuclear gene *CCM1* and the *S. cerevisiae* mitochondrion (Jhuang et al., 2017). The Ccm1 protein is required for the maturation of the *COX1* mRNA in the mitochondrion but its S. *uvarum* version has reduced affinity for the *S. cerevisiae* mRNA. Nucleo-cytoplasmic incompatibility in the opposite direction was also found in cells with incomplete sets of parental chromosomes. The loss of either of the *S. uvarum* nuclear genes *MRS1* and *AIM22* in a hybrid having *S. uvarum* mitotype caused respiration deficiency because their *S. cerevisiae* orthologs cannot serve the *S. uvarum* mitochondrion (Chou et al., 2010). The *S. cerevisiae* Mrs1 protein cannot splice correctly the *S. uvarum COX1* transcript. *AIM22* codes for an enzyme that lipoylates several mitochondrial enzymes. All these incompatibilities prevent sporulation by causing respiration deficiency. Respiration-deficient (petite) cells cannot sporulate (e.g., Kuenzi et al., 1974) because respiration is necessary for the expression of *IME1*, a key gene in the regulation of meiosis and sporulation (Treinin and Simchen, 1993). The incompatibility of the *S. uvarum* mitochondrion with the *S. cerevisiae* nucleus was reinforced by the respiration deficiency of *S. cerevisiae* rho strains supplemented with *S. uvarum* mitochondria (Osuský et al., 1997; Špírek et al., 2000, 2015). However, when examined, the incompatible nuclear genes (alleles) were found recessive. Thus, it is unlikely that these incompatibilities play a significant role in the sterility of the alloploid hybrids because they have also the dominant compatible alleles.

In the present study, we attempt to find correlation between the formation of fertile spores (production of functional gametes) and mitotypes in hybrids of the species *S. cerevisiae* and *S. uvarum*, whose nuclear and mitochondrial genomes are most distantly related in the *Saccharomyces* genus (Kawahara and Imanishi, 2007; Ruan et al., 2017). We selected these species for the experiments because the pioneering studies proposing the involvement of the nucleo-cytoplasmic incompatibility in hybrid sterility (Lee et al., 2008; Chou et al., 2010; Jhuang et al., 2017) were performed with these species and numerous other studies also used these species to investigate the relationship of the nuclear and the mitochondrial genomes (e.g., Osuský et al., 1997; Albertin et al., 2013; Špírek et al., 2015; Verspohl et al., 2018; Li et al., 2019). Besides, we have previously used this species pair to investigate the involvement of the nuclear genome and its changes in the maintenance and break-down of the sterility barrier (Antunovics et al., 2005; Pfliegler et al., 2012).

For the investigation of the mitochondrial genomes in cells of hybrid nuclear genomes, we isolated a large number of cevarum hybrids produced by mass-mating of a *S. cerevisiae* strain and a *S. uvarum* strain marker with complementary auxotrophies. We find high mtDNA diversity in the prototrophic hybrids despite the fact that all hybrids were produced by the same pair of parental strains. Consistent with previous observations made on smaller numbers of hybrids, the majority of the cevarum hybrids inherited the mitochondrial genome from *S. cerevisiae.* Only two hybrids had *S. uvarum* mitochondria but a surprisingly high number of hybrids had recombinant mtDNAs. In spite of the differences in the structure of their mitochondrial genomes, all but one hybrid formed viable but mostly sterile spores and most of them could also produce fertile spores at low frequencies when losing the *MAT*-carrying chromosome of the *S. uvarum* subgenome. During meiosis of the fertile F1 spore clones, the recombinant mitochondrial genomes frequently underwent additional structural modifications resulting in respiration deficiency and concomitant sporulation deficiency in certain F2 spores. The results indicate that the cevarum alloploid nuclear genome is compatible with both parental mitochondrial genomes as well as with their recombinants. Hence, the mitochondrial genome is not directly involved in hybrid sterility but can impair the competitiveness of certain segregants by compromising their respiratory capabilities.

## Materials and Methods

### Strains and Culture Media

The yeast strains used for hybridisation were 10–170 *Saccharomyces cerevisiae MATa leu2* mel^–^ and the homothallic 10–522 *S. uvarum ura3* mel^+^ ts (Antunovics et al., 2005). The strains were maintained at −70 °C and samples of the frozen material were defrosted and propagated on YEA (yeast-extract-agar: 0.5% yeast extract, 1% glucose, 2% agar) or in YEL broth (YEA without agar) to obtain cultures for the experiments. YNBA (yeast-nitrogen-base-agar: 6.7% Bacto Yeast Nitrogen Base, 2% agar) plates supplemented with 2% melibiose were used for sugar assimilation tests. Sporulation was examined on acetate SPA (sporulation agar: 1% K-acetate, 0.1% yeast extract, 0.05% glucose, 2% agar). Hybrids were selected on SMA [synthetic minimal agar medium: 1% glucose, 2% agar, 0.5% (NH_4_)_2_SO_4_, 0.01% KH_2_PO_4_, 0.005% MgSO_4_ and vitamins]. Tests for the presence of auxotrophy markers were performed on SMA supplemented with leucine or uracil or both. The composition of the media was described by Sipiczki and Ferenczy (1977) and Van der Walt and Yarrow (1984).

### Hybridisation and Hybrid Selection

To obtain prototrophic interspecies hybrids the auxotrophic strains were hybridized in two ways. (1) By mass-mating in intersections of line-shaped cultures replica-plated on SMA plates as described by Sipiczki et al. (2018). The strains to be hybridized were streaked on YEA plates (two strains on one plate). After 3 days of incubation the line-shaped cultures were replica-plated onto fresh plates perpendicularly to each other to produce grids of prints in which the parental strains intersected each other. After 5 days of incubation at 20°C, the “grids” were replica-plated onto SMA plates on which the parental strains could not grow. If hybridisation took place, prototrophic colonies appeared at the intersections. Individual colonies were isolated from the intersections, suspended in sterile water and spread onto fresh SMA plates to obtain pure hybrid cultures. (2) By mass-mating of mixed populations of cells and spores of cultures of the auxotrophic strains in YEL using the slightly modified method described in Antunovics et al. (2005). Cultures of the hybridisation partners, grown on the sporulation medium SPA at 20°C for 3 days, were suspended in YEL. After 1.5 h of incubation at room temperature, samples were spread onto SMA plates. The plates were incubated at 30°C for 5 days. The colonies produced were isolated as putative hybrids. The isolates were kept at −70°C to prevent spontaneous segregation.

### Random Spore Analysis and Tetrad Analysis

For random spore analysis samples of the cultures of the isolates grown on plates of sporulation medium for 5 days at room temperature were suspended in Zymolyase-T20 (0.05 mg ml^−1^) solution to dissolve the vegetative cells and lyse the walls of the asci. After incubation at 37°C for 1 h, the suspension was sonicated and aliquots were then spread on YEA plates where the viable spores formed colonies. For tetrad analysis samples of the sporulating cultures were suspended in the Zymolyase-T20 solution and incubated at 37°C for 15 min. Aliquots were then streaked on YEA plates, and four-spored asci were pulled from the streaks by micromanipulation. The asci were dissected and their spores were separated from each other on YEA plates to let them form individual colonies. The colonies produced by the spores (spore clones) were tested for the presence of the auxotrophic markers of the parental strains by replica-plating on SMA plates and SMA plates supplemented with the corresponding compounds.

### Examination of Phenotypic Traits

Sporulation was examined microscopically in cultures smeared on SPA plates and inoculated at 25°C for 5 days. The ability to grow at 37°C or on melibiose and glycerol as carbon sources was examined by drop tests on agar plates. Growth at 37°C was tested on YEA plates. Utilization of glycerol and melibiose was examined on YEA containing 3% glycerol instead of glucose and YNB (yeast nitrogen base) supplemented with 2% melibiose, respectively. 0.2 OD suspensions were prepared from overnight cultures of the strains to be tested and 15-μl samples of the suspensions were dropped on the plates. Results were read after 6 days.

### Electrophoretic Karyotyping

Karyotype analysis was performed as described by Antunovics et al. (2005). Chromosomal DNA was prepared in agarose plugs as described by Nguyen and Gaillardin (1997). Plugs were washed in TE and inserted into wells of 1.1% agarose (Chromosomal grade, Bio-Rad) gel prepared in 0.5 × TBE buffer. The chromosomes were separated by electrophoresis in 0.5 × TBE with a CHEF-Mapper apparatus (Bio-Rad). The running parameters were: 200V, linear ramping from 40 to 120 s for 24 h at 14°C. The chromosomal bands were visualized by staining with ethidium-bromide and destaining in sterile water.

### Mitochondrial DNA Extraction and Restriction Analysis

Mitochondrial DNA was prepared from exponential-phase YEL cultures with the method described by Nguyen et al. (2000) and digested with *Hae*III and *Mbo*I. The fragments were separated by electrophoresis in 0.7% agarose, 0.5 × TBE.

### PCR Amplification and RFLP Analysis of Mitochondrial Genes

Segments of the mitochondrial loci *ATP6*, *COX2*, and *COX3* were amplified with primers described by Albertin et al. (2013). The *ATP6* and *COX3* primers allow differentiating the *S. uvarum* alleles from the *S. cerevisiae* alleles by the PCR product length (the *S. cerevisiae* fragments are larger). The *COX2* primers amplified fragments of identical length from both species, but they could be differentiated by digestion with the restriction endonuclease *Sfc*I. The PCR reactions were carried out with extracted mtDNA as described previously for genomic DNA (Albertin et al., 2013). The introns of the *COX1* gene were amplified with the primers 3L, 3R, 4L, 5R as described by Lopez et al. (2003). PCR fragment sizes were compared by electrophoresis in 1% agarose (w/v), 1 × TBE.

## Results

### Hybridisation

**FIGURE 1:**
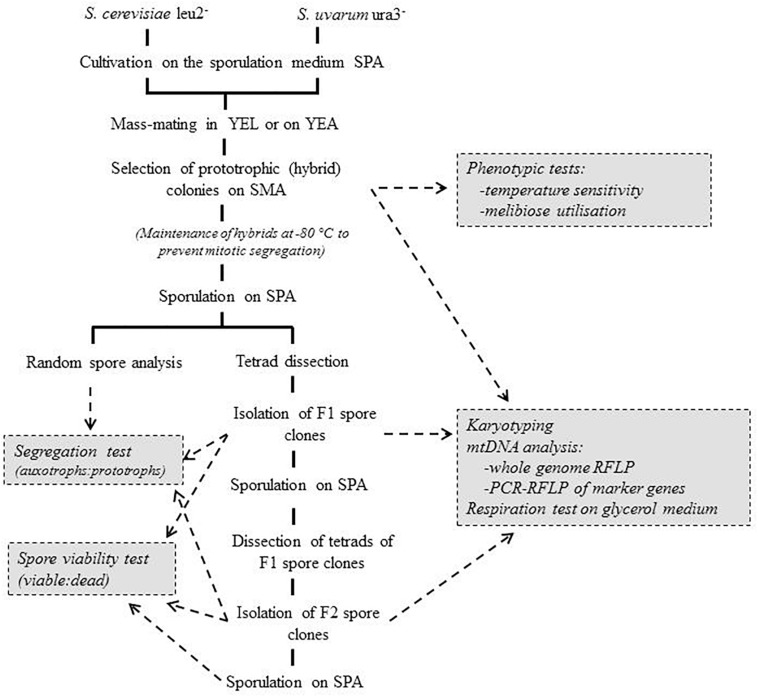
Hybridisation and hybrid analysis.

**FIGURE 2:**
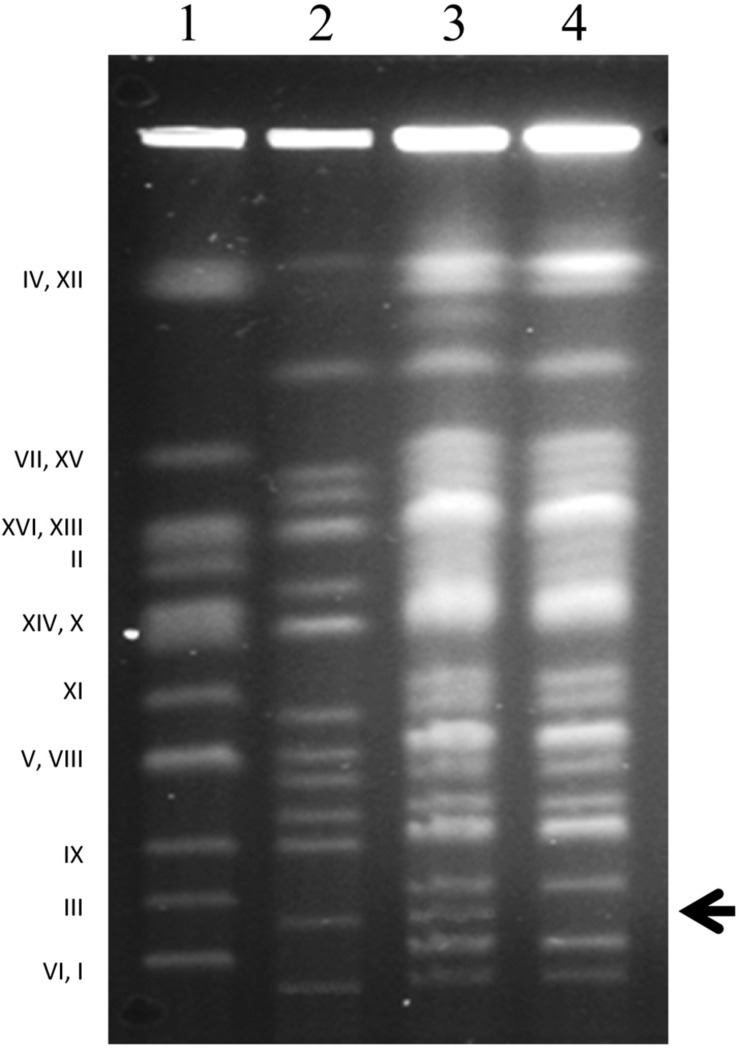
Karyotyping. Chromosomal patterns of *S. cerevisiae* 10–170 **(1)**, *S. uvarum* 10-522 **(2)**, hybrid A4 **(3)** and the fertile F1 spore clone A4.5a of the hybrid A4 **(4)**. Note the missing band in lane 4 (arrowhead), corresponding to the *S. uvarum* chromosome III **(2)**. Designation (numbering) of *S. cerevisiae* chromosomes is shown on the left side.

With two methods of mass-mating of auxotrophic parental strains, 10 (method 1) and 40 (method 2) prototrophic colonies were produced (Figure 1). All proved to have alloploid karyotypes consisting of complete parental sets of chromosomes (example is shown in Figure 2). All grew at 37°C (a trait inherited from the *S. cerevisiae* parent) and assimilated melibiose (a property of the *S. uvarum* parent). In previous studies both traits were found dominant in interspecies hybrids (e.g., Kishimoto, 1994; Zambonelli et al., 1997; Antunovics et al., 2005; Pfliegler et al., 2012). Thus, the prototrophic colonies were interspecies hybrids.

### Allodiploid and Allopolyploid Hybrids

All but one hybrid sporulated (Table 1). The non-sporulating hybrid (A27; category II in Table 1) was most probably allodiploid having single parental sets of chromosomes (haploid subgenomes). *Saccharomyces* cells having single sets of two different species are unable to produce viable spores, due to the failure of the allosyndetic (homeologous) chromosomes of the subgenomes to pair in meiosis-I (the first sterility barrier; Pfliegler et al., 2012).

**TABLE 1:** Sporulation and segregation of hybrids.

			Random spore analysis:			Tetrad analysis: spore	
			segregation of auxotrophic markers^1^			viability and marker segregation	
Category	Strains	Sporulation	leu^–^	ura^–^	leu^–^ ura^–^	viable:dead	auxotroph: prototroph
**Parental strains**							
	10–170	−	leu^–^ mutant	–	–		
	10–522	+	–	ura^–^ mutant	–		
**Hybrids**							
**Method 1**							
I	a1 – a10	+	– or <1% (a5)	–	–	a1 38:2 a2 37:3 a3 39:1 a4 37:3 a5 39:1 a6 40:0 a7 37:3 a8 37:3	a1 4:38 a3 2:38 a5 7:25
**Method 2**							
II	A27	–	–	–	–		
III	A2, A5, A8, A12, A13, A14, A15, A16, A17, A18, A19, A22, A25, A28, A30, A33, A34, A35, A36, A37, A39, A40	+	–	–	–	A2 22:2 A5 16:0	A2 0:22 A5 0:16
IV	A6, A7, A10, A20, A21, A23, A24, A26, A31, A38	+	<1%	–	–	A7 16:4	A7 0:16
V	A1, A9, A11	+	<1%	<1%	–		
VI	A3, A4, A29, A32,	+	>30%	–	–	A3 32:8 A4 10:6 A29 8:0 A32 18:2	A3 25:7 A4 6:4 A29 4:4 A32 8:10

*^1^Note that the overwhelming majority of the auxotrophic segregants were leu^–^, which is consistent with the previous finding that the *S. uvarum* Chr III is lost more frequently than the *S. cerevisiae* Chr III during the meiosis of the allotetraploid cevarum hybrids (Antunovics et al., 2005; Pfliegler et al., 2012).*

The rest of the isolates produced viable spores that germinated and formed colonies (F1 spore clones), albeit with diverse efficiencies. From the viability of the spores we inferred that meiosis took place in their cells. Numerous previous studies demonstrated that alloploid hybrids having genomes higher than diploid can form viable spores (e.g., Banno and Kaneko, 1989; Greig et al., 2002; Pfliegler et al., 2012; Karanyicz et al., 2017; García-Ríos et al., 2019). As meiosis necessitates efficient chromosome pairing, each chromosome in these isolates must have had an autosyndetic (homologous) partner to pair with. Thus, these hybrids were allopolyploids (e.g., most probably allotetraploids or allotriploids). Allotetraploids have two sets of chromosomes in both subgenomes, hence each chromosome has an autosyndetic partner for pairing. Allotriploids can also produce viable spores because their cells have two sets of the chromosomes of one of the parents which can pair within their subgenome. As meiosis produces four ascospores, the spores of a tetraploid yeast cell are diploid (Leupold, 1956); in the case of allotetraploid meiosis the spores are allodiploid. The spores of the allotriploid cells can have haploid sets of chromosomes from the diploid subgenome and randomly distributed chromosomes of the haploid subgenome (alloaneuploid spores). To examine the genomes of the viable spores, we karyotyped certain spore-derived colonies (spore clones) (Figure 1). Almost all had complete sets of chromosomal bands of both parents in their karyotypes, demonstrating that they had at least allodiploid genomes, and the genomes of the hybrids were tetraploid rather than triploid. The exceptions lacked the band corresponding to Chr III of *S. uvarum* (Figure 2). The different efficiencies of the isolates to produce viable spores, however, hint at the possibility that certain hybrids had allotriploid genomes or their cultures were heterogeneous in ploidy, containing also cells with ploidies different from allotetraploidy. We considered the asci containing four viable spores (complete tetrads) products of allotetraploid cells.

### Stable and Segregating Hybrids

The F1 spore clones that possessed all types of chromosomes of both parents were prototrophic, most probably because they were heterozygous for both recessive auxotrophic markers (leu1^–^ and ura3^–^) used for hybrid selection. The majority of the hybrids produced only prototrophic spores (categories I and III in Table 1) which corroborates the notion that the allotetraploid meiosis produces allodiploid spores (heterozygous for the markers). However, 17 hybrids segregated. 14 hybrids formed less than 1% (categories IV and V in Table 1 and one hybrid in category I) and 4 hybrids produced more than 30 % auxotrophic spores in random spore analysis (category VI, Table 1). The loss of the wild-type alleles of these markers implies that meiosis failed to transmit copies of all chromosomes into all spores. The auxotrophic spores did not receive copies of the parental chromosomes carrying the wild-type alleles of the markers. Remarkably, most segregating hybrids lost the *LEU1* allele of *S. uvarum* (categories IV and VI, Table 1). Only 3 hybrids produced both leu^–^ and ura^–^ segregants (category V, Table 1).

### Loss of Sterility by Meiotic Malsegregation of *S. uvarum* Chr. III (Chr. 2)

To verify the association of the loss of marker heterozygosity with the loss of a chromosome, we subjected certain F1 spore clones to karyotyping (Figure 1). The leu^–^ clones lacked the band corresponding to Chr. III of *S. uvarum* (designated Chr. 2 in Nguyen et al., 2000) (Figure 2), whereas the prototrophs had complete alloploid karyotypes. As Chr. III carries also the *MAT* locus, we supposed that the loss of the wild-type *LEU1* allele was entailed with the simultaneous loss of *MAT* heterozygosity. In a previous study we noticed that the loss of *MAT* heterozygosity reactivated mating-type switching and the mating program repressed in the *MATa/MATalpha* hybrid cells (Pfliegler et al., 2012). Because of these changes, the vegetative descendants of the aneuploid spore (nullisomic for one parental Chr. III) could switch their mating type and conjugate with each other to double their genomes (resulting in incomplete allotetraploids nullisomic for one parental Chr. III). In that study, the outcomes of the process were cells with restored fertility that formed viable F2 spores. To find out if the loss of *MAT* heterozygosity by the loss of the *S. uvarum* chromosome Chr. III had a similar effect in the hybrids produced in this study, we isolated F2 spores from certain asci of the F1 leu^–^ spore clones of the four unstable hybrids (Figure 1). The isolated spores were viable and formed colonies (F2 spore clones) (Table 2), which clearly demonstrated that the loss of this chromosome broke down the sterility barrier.

**TABLE 2:** Restoration of fertility (break-down of the sterility barrier) in unstable hybrids.

	Spore clone			
Hybrid	F1	F2^1^	Auxotrophy^2^	Sporulation
A3			P	+
	1a		P	+
	1b		leu^–^	+
		1a,b,c,d^3^	leu^–^	+,+,+,+
		2a,b,c,d^3^	leu^–^	+,+,+,+
		3a,b,c,d^3^	leu^–^	+,+,+,+
		4a,b,c,d^3^	leu^–^	+,+,+,+
	1c		leu^–^	+
	1d		P	+
A4			P	+
	5a		leu^–^	+
		1a,b,c,d^4^	leu^–^	+,+,+,+
		2a,b,d^4^	leu^–^	+,+,+
		3b,c,d^4^	leu^–^	+,+,+
	5b		P	+
	5c		P	+
	5d		leu^–^	+
A29			P	+
	1a		P	+
	1b		P	+
	1c		leu^–^	+
	1d		leu^–^	+
A32				
	1a		leu^–^	+
		1a,b,c,d^5^	leu^–^	+,+,+,+
		2a,b,c,d^5^	leu^–^	+,+,+,+
		3a,b,c,d^5^	leu^–^	+,+,+,+
	1b		leu^–^	+
	1c		P	+
	1d		P	+

*Example tetrads are shown. ^1^Clones listed in one raw are sister clones produced by spores of the same ascus (tetrad). ^2^P stands for prototroph. ^3^F2 spore clones formed by spores of the F1 spore clone 1b of hybrid A3. ^4^F2 spore clones formed by spores of the F1 spore clone 5a of hybrid A4. ^5^F2 spore clones formed by spores of the F1 spore clone 1a of hybrid A32.*

### Whole-Genome Mitotypes of Hybrids

**FIGURE 3:**
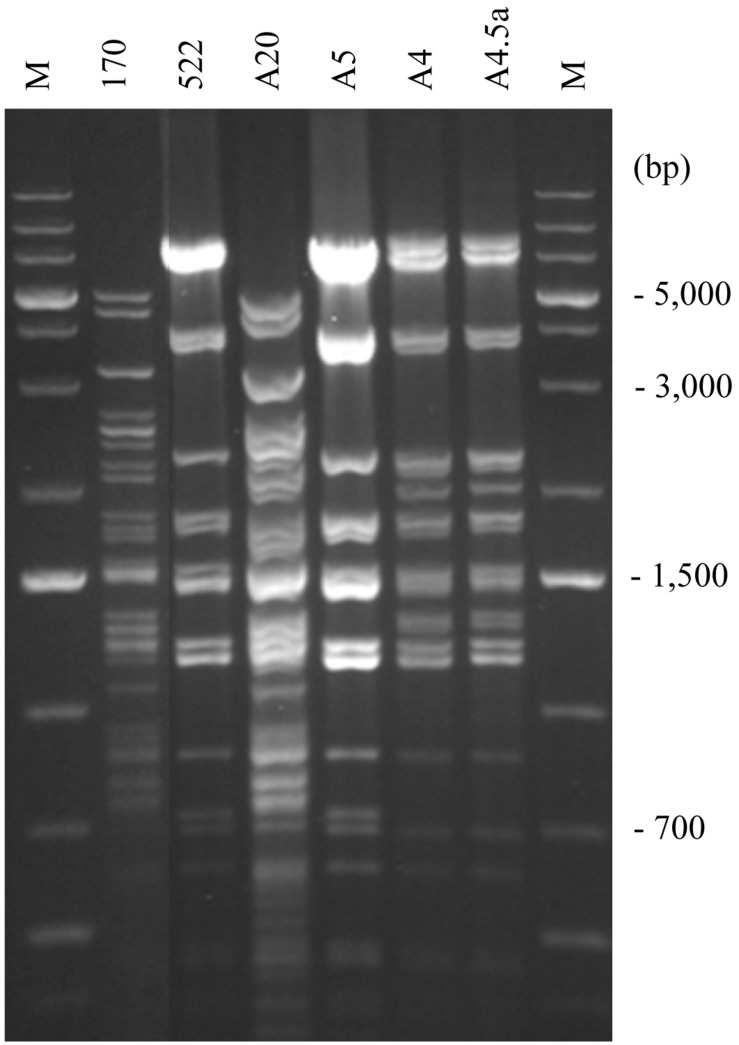
RFLP analysis of mitochondrial genomes. *S. cerevisiae* 10-170 (170); *S. uvarum* 10-522 (522); *S*. *cerevisiae* mitotype in the hybrid A20 (A20); *S. uvarum* mitotype in the hybrid A5 (A5); R7-type recombinant mitotype in the hybrid A4 (A4); one of the F1 spore clones of the hybrid A4 (A4.5a); M, size ladder.

As all karyotyped hybrids had complete sets of parental chromosomes, we asked whether they also had the mitochondrial genomes of both parents. For gross comparison of the structures of the mitochondrial genomes, mtDNA was isolated from the cells and digested with restriction endonucleases (Figure 1). After separation of the fragments by gel electrophoresis, we compared the banding patterns of the hybrids with each other and with those of the parental strains (Figure 3 and Table 3). The banding patterns of the parental strains were clearly different; 30 hybrids had *S. cerevisiae*-type patterns whereas only 2 hybrids had *S. uvarum*-type mtDNA patterns. 18 hybrids differed from both parents. One of these hybrids (a3) turned out to be a mixed clone consisting of a subpopulation showing the *S. cerevisiae* pattern and a subpopulation having a non-parental pattern. The latter subclone and the rest of the group had various combinations of bands corresponding in size to certain parental bands. None of these patterns had all bands of both parents and six patterns contained extra bands not seen in the parents. Altogether eight non-parental band combinations (R1–R8 in Table 3) could be distinguished.

**TABLE 3:** Mitotypes of hybrids and glycerol utilization as a carbon source.

					Allels of			Growth on glycerol at	
						
					COX1				
Category of nuclear nuclear genome^1^	Category of mitotypes^2^	Strains	Whole-genome RFLP^3^	ATP6	COX1 intron^4^	COX2^5^	COX3	25 °C	37 °C
Parental strains									
	C	10-170	C	C	C	C	C	+	+
	U	10-522	U	U	U	U	U	+	−
Hybrids									
Method 1									
I	C	a2, a4, a5, a6, a7, a9, a10	C					+	+
	I-r1	a1	R1	C	C	Rc1	C	+	+
	I-r2	a3	C					+	+
			R7	U	−	Rc2	U	+	+
	I-r3	a8	R2	U	C	Rc1	U	+	-
Method 2									
II	II-r1	A27	R3	U	C	C	U	-	-
III	C	A12, A13, A14, A16, A17, A18, A19, A22, A25, A28, A30, A34	C					+	+
	U	A5	U					+	+
	III-r1	A37	R4	C	C	U	U	+	(+)
	III-r2	A2	R4	C	C	U	U	+	+
	III-r3	A8	R5	C	C	U	U	+	+
	III-r4	A15	R6	C	C	U	U	+	+
	III-r5	A33	R5	C	C	U	U	+	−
	III-r6	A35	R7	U	C	U	U	+	−
	III-r7	A36	R8	C	C	U	U	+	+
	III-r8	A39, A40	R7	U	Ri1	U	U	+	-
IV	C	A7, A10, A20, A23, A26, A31	C					+	+
	IV-r1	A6, A38	R6	C	Ri2	U	U	+	+
	IV-r2	A21	R2	U	C	U	U	+	−
	IV-r3	A24	R2	U	C	U	U	+	−
V	C	A1, A9, A11	C					+	+
VI	C	A29, A32	C					+	+
	U	A3	U					+	+
	VI-r1	A4	R7	U	C	U	U	+	−

*^1^For description of categories, see Table 1. ^2^Based on all sets of results shown in the table. ^3^RFLP pattern types (for examples, see Figure 3): C, *S. cerevisiae* parental pattern; U, *S. uvarum* parental pattern. Banding patterns (bands larger than 700 bp, starting the order with the largest band): C: pqrstuvw U: ABCDEF R1: pqrs.uvw R2:..r..A.... ABCD.F 1 and 2 (new bands)R3: r R4: ..r R5: ..r..**u**. R6: .qrs.uvw ABCDEF ABCDEF ABCDEF A 1 and 2 (new bands) 2 (new band) 2 (new band) R7:. qr..u. R8: ABC.EF ABCD.F 2 (new band) 2 (new band)^4^Ri1 and Ri2 are recombinant *COX1* patterns. ^5^Rc1 and Rc2 are recombinant *COX2* patterns.*

### Mosaic (Chimeric) mtDNA in Hybrids of Non-parental RFLP Patterns

**FIGURE 4:**
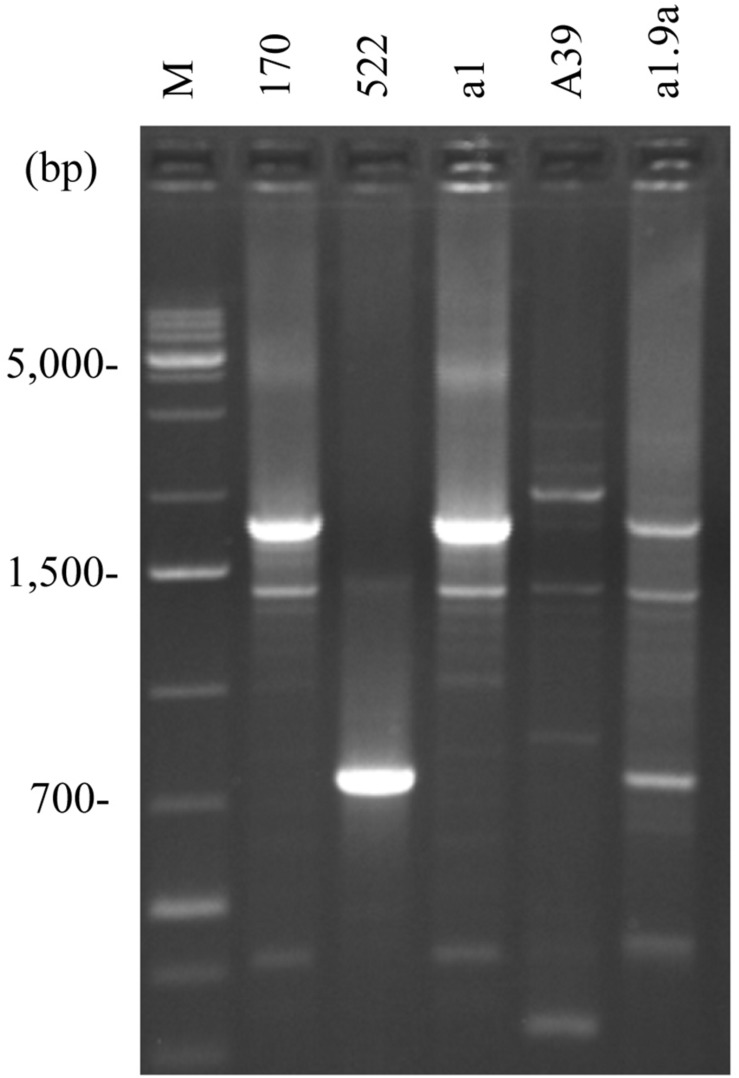
Intron diversity in *COX1*. *S. cerevisiae* 10–170 (170); *S. uvarum* 10–522 (522); hybrid a1 with *S. cerevisiae* pattern (a1); hybrid A39 with Ri1-type recombinant pattern (A39); F1 spore clone of the hybrid a1 showing Ri3-type recombinant pattern (a1.9a); M, size ladder.

**FIGURE 5:**
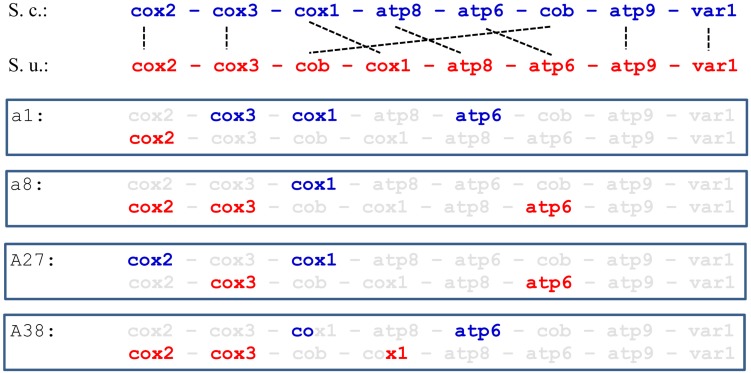
Structure of mitochondrial genomes. *S. cerevisiae* 170 (S. c.), *S. uvarum* 522 (S. u.) and four hybrid strains that have recombinant mitochondrial genomes. Markers found are bold.

Non-parental RFLP patterns can be generated either by rearrangement in one or the other parental mtDNA or by their recombination at conjugation or thereafter, during the vegetative propagation of the hybrid cells. As no drastic genome-size changes were noticed, the more probable mode of interaction was the exchange of segments between the parental mitochondrial genomes via recombination resulting in mosaic (chimeric) genome structures. To examine this possibility, we set out to determine the origin of the loci *ATP6*, *COX1*, *COX2*, and *COX3* (Figure 1). These markers were used in previous studies for monitoring the transmission and recombination of mtDNA in yeast hybridisation experiments (e.g., Rainieri et al., 2008; Solieri et al., 2008; Garcia Sanchez et al., 2012; Albertin et al., 2013; Špírek et al., 2015; Verspohl et al., 2018). By amplification with locus-specific primers and restriction analysis of the amplicons, the *S. cerevisiae* and *S. uvarum ATP6*, *COX2*, and *COX3* genes can be distinguished (Albertin et al., 2013). All hybrids showing non-parental RFLP patterns had non-recombinant, parental-type *ATP6* and *COX3* genes (Table 3). Equal numbers of hybrids had *S. cerevisiae*-type and *S. uvarum*-type *ATP6* genes. All but one of these hybrids had the *COX3* gene from *S. uvarum*. Fourteen hybrids had the *S. uvarum* version of *COX2*, three hybrids had recombinant *COX2* and only one hybrid had this gene from *S. cerevisiae*. The origin of the *COX1* genes was examined with two pairs of primers. In the four-primer amplification, the parental strains differed in the number and size of the bands. Two hybrids had recombinant *COX1* banding patterns; the rest of the hybrids had *S. cerevisiae* patterns (Figure 4). The analysis of the four loci reinforced that the mitochondrial genomes of non-parental RFLP patterns were recombinants consisting of mosaics of the parental genomes. Recombination took place both in the intergenic regions and within genes (four examples of chimeric genomes are shown in Figure 5).

### Nuclear Genome Instability and Mitotypes

The hybrid defective in sporulation (A27) had a unique chimeric mtDNA, but as long as more non-sporulating hybrids are not analyzed, it cannot be inferred that this mitochondrial genome structure causes the sporulation deficiency. Viable spores were produced in hybrids with either type of parental mitochondrial genomes and with their various chimeric versions (Table 3). No correlation was found between the mitochondrial genomes and the instability of the nuclear genomes either. The four hybrids of category VI that formed auxotrophic spores at high frequencies had three types of mitochondrial genomes, two of which were parental (Table 2). The three hybrids, which produced both leu^–^ and ura^–^ spores (category V), had *S. cerevisiae*-type mitochondria, but 37 hybrids belonging to other categories also had this parental mitotype.

### Mitotypes and Glycerol Utilization

*Saccharomyces* cells gain energy from glucose by glycolysis leading to pyruvate, which is then converted either to ethanol, lactic acid (fermentation) or CO_2_ (respiration). The latter process requires oxygen and active mitochondria. Cells having functional mitochondria can grow on non-fermentable carbon sources such as glycerol if sufficient amount of oxygen is available. Strains unable to grow on glycerol are considered respiration deficient (Nagai et al., 1961). As the hybrids had diverse mitochondrial genome structures, we tested them for growth on glycerol (Table 3 and Supplementary Figure 1). Both parents and all but one hybrid grew on glycerol as the only carbon source at 25°C. The exception was the non-sporulating hybrid A27. At 37°C, a temperature restrictive for *S. uvarum*, the *S. uvarum* parent and certain hybrids did not grow on glycerol or their growth was retarded, indicating that their respiration was temperature sensitive. 37°C inhibited very severely the growth of the III-type hybrids A35, A37, A39, A40, the IV-type hybrids 21, 24 and the VI-type hybrid A4. The III-type hybrid A33 was also retarded in growth at 37°C but less drastically than the previous hybrids. Remarkably, the respiration of the other hybrids of these categories was not sensitive to 37°C. A common feature of all hybrids showing temperature-sensitive respiration was the lack of the *S. cerevisiae* alleles of *ATP6*, *COX2*, and *COX3*. It is pertinent to mention here that the proteins encoded by these genes in the *S. cerevisiae* and *S. uvarum* type strains are less similar than the Cox1 proteins. When comparing the amino acid sequences (EMBOSS Matcher^1^), we found 93.8, 96.8, and 97.8% interspecies sequence identity for Atp6 (accession numbers: AOT85185.1 and AOT85145.1), Cox2 (accession numbers: AOT85175.1 and AOT85135.1) and Cox3 (accession numbers: AOT85177.1 and AOT85137.1), respectively, whereas the Cox1 proteins were 98.5% identical (accession numbers: AOT85178.1 and AOT85141.1) in sequence. The higher similarity between the Cox1 proteins suggests that the Cox1 orthologs have better interchangeable compatibility for complex IV.

### Meiotic Segregation of Recombinant Mitotypes

**FIGURE 6:**
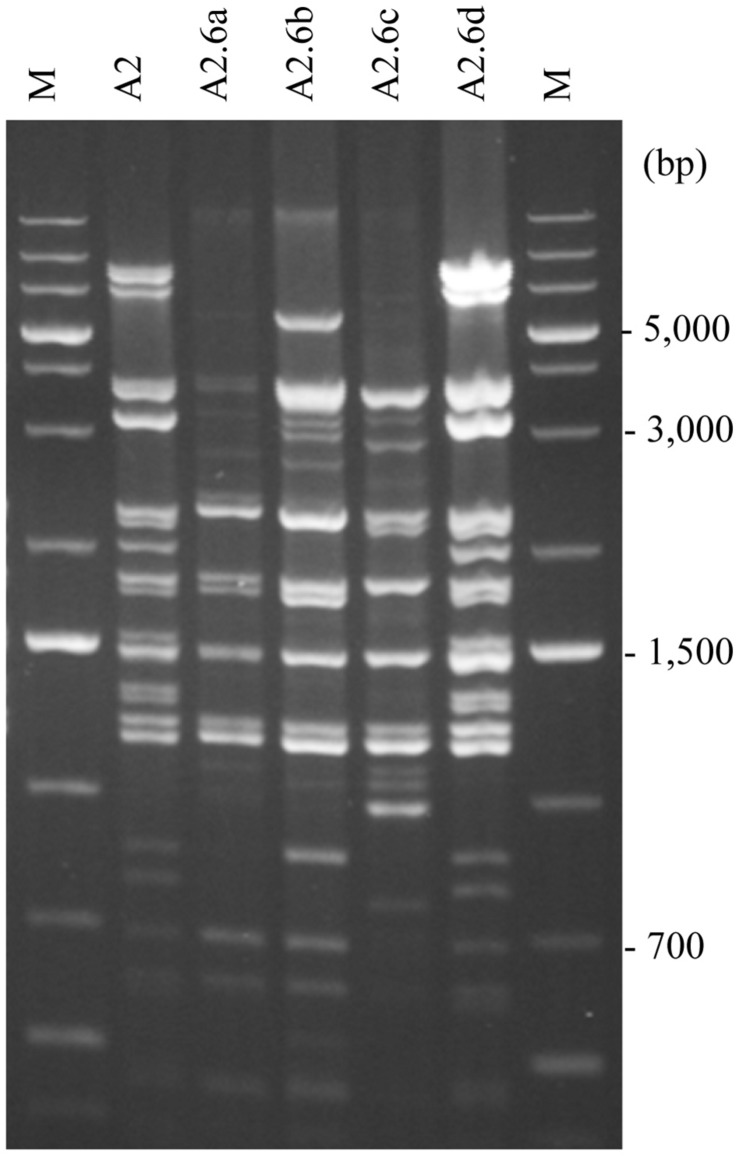
Meiotic segregation of mitotypes. RFLP patterns of the hybrid A2 and its F1 spore clones isolated from a complete tetrad. M, size ladder.

To investigate the stability of the mitochondrial genomes at meiotic division found in previous studies to be prone to destabilize the allotetraploid nuclear genome (for a review, see Sipiczki, 2018), spores were isolated from the asci of certain hybrids (Figure 1). When tested for auxotrophy, the spores of the complete tetrads (four viable spores in the ascus) usually formed prototrophic F1 spore clones or prototrophic and leu^–^ spore clones mostly in 1:1 ratio. The mitochondrial genomes of the clones were then examined with whole-genome RFLP analysis (Figure 6) and the amplification of the genes *ATP6*, *COX1*, *COX2*, and *COX3* (examples are shown in Table 4). The hybrids having parental-type mitochondrial genomes usually transmitted their mtDNA unchanged into the ascospores. A5 was an exception because its *S. uvarum*-type mitochondrial genome underwent changes in certain spores. In contrast, the hybrids which had recombinant mitochondrial genomes produced F1 spore clones with additional modifications in the structure of the mtDNA at higher frequency. In the whole-genome RFLP patterns of their mitochondria, bands were missing or replaced by new bands. These changes were occasionally associated with the loss of one or the other of the genes monitored by PCR-amplification and/or with rearrangements in the intron structure of *COX1*. However, no correlation was observed between the segregation of the nuclear genome and the changes in the mitochondrial genomes. The loss of the *S. uvarum LEU1* allele (by the loss of the *S. uvarum* chromosome III) did not affect the stability of the mitochondrial genome. As none of the hybrids were heterozygous for any of the examined loci, the changes of their mitotypes during meiosis-sporulation can be attributed to intragenomic rearrangements rather than to recombination between parental mtDNAs.

**TABLE 4:** Meiotic segregation.

						Allelles of				Growth on/at		
						Mitotype		COX1				
Hybrid	Spore clone		Auxotrophy^1^	Sporulation	RFLP^2^	ATP6	intron	COX2	COX3	2 % glucose	glycerol	
									
	F1	F2								37 °C	25 °C	37 °C
a1			P	+	R1	C	C	Rc1	C	+	+	+
	7a		P	+	R1					+	+	−
	7b		P	<1%	R1					+	+	+
	7c		P	<1%	R1					+	+	+
	7d		P	−	X:qrsuv	−	C	Rc1	−	+	+	+
	9a		leu	−	X:pqrstuw	C	Ri3	Rc1	−	−	−	−
	9b		leu	−	X:qrsuvw	C	C	Rc1	−	−	−	−
	9c		leu	−	X:qrsuvw	−	−	Rc1	−	+	−	−
	9d		leu	−	X:qu(w)	−	−	Rc1	−	+	+	−
a3			P	+	C+R7	U	−	Rc1	U	+	+	+
	1a		P	+	R7					+	+	−
	1b		leu	−	X: EF	U	Ri4	Rc1	−	+	−	−
	1c		P	+	R7					+	+	−
	1d		P	+	R7					+	+	−
	4a		P	−	X: CEF (w/2)	U	Ri4	Rc1	−	+	−	−
	4b		P	+	X:BCEF					+	+	−
	4c		P	+	R7					+	+	−
	4d		P	+	R7					+	+	−
	8a		leu	−	X: DEF	U	Ri4	Rc1	−	+	−	−
	8b		P	+	R7					+	+	−
	8c		P	+	R7					+	+	−
	8d		P	+	R7					+	+	−
A2			P		R4	C	C	U	U	+	+	+
	3a		P	+	R4					+	+	−
	3c		P	−	X: (C)EF	C	C	U	−	+	−	−
	3d		P	<1%	R4					+	+	−
	5a		P	<1%	R4					+	+	−
	5b		P	+	R4					+	+	−
	5c		P	−	R4					+	+	−
	5d		P	+	R4					+	+	−
	6a		P	−	X: (CD)EF(r)+	C	−	U	−	+	−	−
	6b		P	−	X: CDEF r+	C	C	U	−	(+)	−	−
	6c		P	−	X: CDEF r+	C	C	U	−	+	−	−
	6d		P	−	R4	C	C	U	U	−	+	−
A3			P	+	U	U	U	U	U	+	+	+
	1a		P	+	U					+	+	+
	1b		leu	+	U					+	+	−
		1a,b,c,d	leu	+							+	
		2a,b,c,d	leu	+							+	
		3a,b,c,d	leu	+							+	
		4a,b,c,d	leu	+						+	+	
	1c		leu	+	U					+	+	−
	1d		P	+	U					+	+	−
A4			P	+	R7	U	C	U	U	+	+	−
	5a		leu	+	R7					+	+	−
		1a,b,c,d	leu	+								
		2a,b,d	leu	+								
		3b,c,d	leu	+								
	5b		P	+	R7					+	+	−
	5c		P	+	R7					+	+	−
	5d		leu	+	R7					+	+	−
A5					U	U	U	U	U	+	+	+
	2a		P	+	U					+	+	+
	2b		P	+	U					+	+	+
	2c		P	+	U					+	+	+
	2d		P	−	U					+	+	+
	4a		P	−	U					+	+	−
	4b		P	−	X: ABCD(EF)+	U	U	U	−	+	−	−
	4c		P	−	X: BDEF	U	U	U	−	+	−	−
	4d		P	−	X: ABCD(EF)+	U	U	U	U	+	−	−
A29			P	+	C	C	C	C	C	+	+	+
	1a		P	+	C					+	+	+
	1b		P	+	C					+	+	+
	1c		leu	+	C					+	+	+
	1d		leu	+	C					+	+	+
A32					C	C	C	C	C	+	+	+
	1a		leu	+	C					+	+	+
		1a,b,c,d	leu	+								
		2a,b,c,d	leu	+								
		3a,b,c,d	leu	+								
	1b		leu	+	C					+	+	+
	1c		P	+	C					+	+	+
	1d		P	+	C					+	+	+

*^1^P, prototrophic. ^2^C, *S. cerevisiae* parental pattern; U, *S. uvarum* parental pattern; R, recombinant pattern; X: rearranged pattern with reduced numbers of parental bands (retained bands are shown) and novel bands; +: new band(s) not seen in the parental patter (Figure captures).*

## Discussion

### Hybrid Sterility and the Break-Down of the Second Sterility Barrier by Loss of the *MAT*-Carrying Chromosome of the *S. uvarum* Subgenome (Loss of *MAT* Heterozygosity)

By two methods based on natural mating of mutants with complementary auxotrophy markers, 50 prototrophic cevarum (*S. cerevisiae x S. uvarum*) hybrids were isolated. One hybrid did not form spores in spite of having complete sets of parental chromosomes. Its sporulation deficiency can be attributed to the first part of the postzygotic double sterility barrier. This barrier operates in allodiploids and is attributable to the inability of the allosyndetic (homeologous) chromosomes of the subgenomes to pair during prophase-I of meiosis (Pfliegler et al., 2012).

The rest of the hybrids produced viable spores, indicating that the first sterility barrier was ineffective in their cells. The spores germinated and developed clones of vegetatively propagating cells (F1 spore clones). Previous studies revealed that allopolyploid (e.g. allotetraploid) *Saccharomyces* hybrids produce viable ascospores (Banno and Kaneko, 1989; Greig et al., 2002; Sebastiani et al., 2002; Antunovics et al., 2005; Pfliegler et al., 2012) because chromosome pairing becomes can take place within the duplicated subgenomes (Karanyicz et al., 2017). Since the subgenomes of the allotetraploids are autodiploid, the process is called autodiploidisation of the allotetraploid meiosis. The phenomenon was first observed in allotetraploid plant hybrids (for a review, see Ramsey and Schemske, 2002) and recently described also in *Saccharomyces* interspecies hybrids (Karanyicz et al., 2017). From the viability of their spores we inferred, that the sporulation-proficient hybrids produced in this study had allotetraploid genomes. However, the production of viable spores does not imply that genome duplication overrides the sterility barrier. In a previous study (Pfliegler et al., 2012) we found that the cells of the F1 spore clones were sterile. Being allodiploid, their cells could neither conjugate nor sporulate due to the repression of the genes of the mating (fertilization) process by *MATa/MATalpha* heterozygosity and the inability of the allosyndetic chromosomes to pair in prophase-I (the second sterility barrier). Consistent with our earlier observations, the F1 spore clones of most sporulation-proficient hybrids of this study failed to produce F2 spore clones. Hence, these hybrids were also sterile.

As the inability of the F1 spore clones to mate is the consequence of the interaction between *MATa* and *MATalpha*, the loss of *MAT* heterozygosity by malsegregation of the *MAT*-carrying chromosomes of one of the partner genomes during tetraploid meiosis can abolish the second sterility barrier (Pfliegler et al., 2012). Being diplontic organisms, the *Saccharomyces* strains have a meiotic apparatus optimal for handling diploid sets of chromosomes. When the ploidy increases, the fidelity of meiosis decreases (Loidl, 1995). If the allotetraploid meiotic cell fails to transfer copies of both parental chromosomes carrying the *MAT* locus into each spore, alloaneuploid spores are formed which have only one *MAT* allele. The vegetative descendants of these spores can switch their mating types and can mate with other cells of the clone. In our hybrids such alloaneuploids could be identified as leu^–^ spores because the *LEU1* gene is on the *MAT*-carrying chromosome in both parental species, and the *S. uvarum* subgenome had the wild-type allele of the gene. These spore clones lacked the corresponding *S. uvarum* chromosomal band in their karyotypes. 34% of the hybrids formed leu^–^ spores at low frequency, but four of them produced more than 30%. These results are consistent with those of previous works which showed that the synthetic polyploid cevarum hybrids occasionally produce fertile spores by losing the *MAT*-carrying chromosome of the *S. uvarum* subgenome (Antunovics et al., 2005; Pfliegler et al., 2012).

### Hybrid Homoplasmy

Consistent with previous reports (e.g., Antunovics et al., 2005; Solieri et al., 2008; Albertin et al., 2013; Karanyicz et al., 2017; Verspohl et al., 2018), all but one hybrid constructed in this study were homoplasmic. 64% of the hybrids had one or the other clearly distinguishable parental-type mtDNA RFLP pattern. The rest had non-parental RFLP patterns and various combinations of the parental orthologs of genes used as markers in the analysis. None of these hybrids had complete parental sets of RFLP bands and both parental versions of the marker genes. The only exception, hybrid a3 formed a mixed culture consisting of cells having *S. cerevisiae* mtDNA and cells having recombinant mtDNA.

As the cells of different *Saccharomyces* species conjugate in much the same way as the *S. cerevisiae* cells, it can be assumed that the transmission of parental mitochondria into the zygotes also takes place similarly in the intra- and the interspecies conjugations. When *S. cerevisiae* cells conjugate, the zygote contains the mitochondria of both parental cells (reviewed in Birky, 1978). If their mitochondria are different, the zygote is heteroplasmic. After karyogamy, the zygote produces a bud that receives the fused nucleus and copies of both types of mitochondrial genomes. When the bud divides, its vegetative descendants inherit both mitochondrial genomes (biparental inheritance). However, the heteroplasmic state is only transient. After a few rounds of division, the clone becomes homoplasmic (Dujon et al., 1974; Birky et al., 1978a). The results of a recent study of Albertin et al. (2013) suggests that gradual transition from heteroplasmy to homoplasmy takes place also in the interspecies hybrids. They found two types of mitochondria in certain cevarum hybrids after 20 rounds of vegetative divisions but only one type after 80 generations. But they did not examine the possibility that the culture at the 20th generation could have been a mixture of different homoplasmic subpopulations, from which one overgrew the other subpopulations by the 80th generation. Our hybrid a3 indicates that the transition from heteroplasmy to homoplasmy can take place in more than one way within a hybrid culture, converting it in a mixture of different homoplasmic subpopulations.

The mechanism of the heteroplasmy-to-homoplasmy transition is not fully understood but appears to be influenced in *S. cerevisiae* intraspecies hybrids by several factors such as the position of the bud arising from the zygote (Strausberg and Perlman, 1978; Zinn et al., 1987), difference in the number of mitochondria in the conjugating cells, different copy numbers of the parental mtDNA molecules (Fangman et al., 1989), different density and activity of replication origins in the parental mtDNAs (de Zamaroczy et al., 1981), different level of membrane polarization (due to decreased respiratory chain activity) and the degradation of the less polarized mitochondria by autophagy (Karavaeva et al., 2017). The process can start already in the zygote because heteroplasmic yeast zygotes have upregulated levels of mitochondrial degradation by autophagy (Karavaeva et al., 2017). The model based on the observation that mtDNA replicates in a rolling-circle model on randomly selected mtDNA molecules into the buds can explain the establishment of homoplasmy, but does not explain how the hybrid clone becomes uniformly homoplasmic (Ling and Shibata, 2004).

Similar mechanisms can be assumed to operate in the cevarum hybrids as well. Nevertheless, from the high proportion of hybrids with recombinant mitotypes we infer that both parental mitochondria were present in the cevarum zygotes which then fused to allow their DNAs to recombine. Further we suppose that the subpopulations having recombinant mitochondrial genomes and being more fit in the culturing conditions outcompeted the subpopulations which had parental or less favorable recombinant mitochondrial genomes. The structural diversity of the recombinant mitochondrial genomes can be ascribed to recombination events at random positions. The intensifying research into yeast mitochondrial biology (e.g., Wu et al., 2015; Higuchi-Sanabria et al., 2016; Pernice et al., 2016, 2018; Chakraborty et al., 2017; Guaragnella et al., 2018; Prevost et al., 2018; Ling et al., 2019; Dhar et al., 2019; Peris et al., 2019) will surely provide novel tools for the deciphering of the complexity of the mechanisms underlying the transmission of the mitochondrial genomes into the hybrid bud formed on the zygote and their subsequent evolution.

### Preferential Transmission of the *S. cerevisiae* Mitochondrial Genome (Mitotype) to Hybrids

Sixty six percent of the hybrids had parental mitotypes, either from *S. cerevisiae* or from *S. uvarum*, demonstrating that neither parental mitochondrial genome is incompatible with the alloploid nuclear genome. Although both types of hybrids were viable and propagated equally well on glucose-containing media, only two of them had *S. uvarum* mitotypes. Previous studies including our own work also observed preferential transmission of *S. cerevisiae* mitochondria to cevarum hybrids (e.g., Marinoni et al., 1999; Delneri et al., 2003; Antunovics et al., 2005; Lee et al., 2008; Solieri et al., 2008; Albertin et al., 2013; Li and Fay, 2017; Origone et al., 2018). Marinoni et al. (1999) found that cevarum hybrids had *S. uvarum* mitochondria only when the *S. cerevisiae* parent was petite (did not contain functional mitochondrial genome). Other authors (Solieri et al., 2008; Verspohl et al., 2018) found that the hybrids obtained from mating of the same parental strains always had the same parental mitotype but hybrids of other strains of the species could have mitochondria from the other species. In our hands, the hybrids produced by the same pair of parental strains were heterogeneous. 62% had *S. cerevisiae* mitotypes, 4% had *S. uvarum* mitotypes, and 34% had diverse recombinant mitotypes. The observed diversity indicates that neither parent can exclusively determine the hybrid mitotype, although the *S. cerevisiae* parent is more successful in transferring its mitochondrial genome.

The mating of yeast cells is an isogamic process, resulting in a heteroplasmic zygote containing all mitochondria of the conjugating cells (see above). Why is then the *S. cerevisiae* mitochondrial genome preferentially transmitted to the hybrid cells? Since the underlying mechanism is not known, we propose two alternative models. One possibility is that certain components of the mechanism controlling the replication of mtDNA promote the transmission of copies of the *S. cerevisiae* mtDNA into the first bud formed on the zygote. Its preferential transmission could be due to its faster replication. Cardazzo et al. (1998) observed greater mtDNA replication capacity in the *S. cerevisiae* mitochondrial genome than in the *S. uvarum* mitochondrial genome. The difference was attributed to the different numbers of replication origins in the two species. The *S. cerevisiae* mitochondrial genome was estimated to have 7 or 8 replication origins whereas the *S. bayanus* (*uvarum*) mitochondrial genome contained only 4 origins (de Zamaroczy et al., 1981; Cardazzo et al., 1998; Piskur et al., 1998). The alternative possibility is that the *S. cerevisiae* mtDNA is not replicated faster but makes the hybrid cells somewhat more competitive compared to the hybrid cells possessing *S. uvarum* mtDNA or recombinant mitochondrial genomes. In this model we assume that the bud of the zygote produces heteroplasmic hybrid cells, but these cells then segregate (much like the intraspecies *S. cerevisiae* hybrids; Birky et al., 1978b). Their culture becomes a mixture of homoplasmic subpopulations having parental and recombinant mitotypes. Those having *S. cerevisiae* mitotypes may have a better chance to overgrow the other subpopulations on the medium used for hybrid selection. We found two types of mtDNA in the culture of only one hybrid characterized in this study.

### Recombination of Parental Mitochondria in the Hybrids

One third of the hybrids had non-parental (recombinant) mitotypes. This recombination rate is much higher than those detected previously in cevarum hybrids. da Silva et al. (2015) found no recombinant mitochondria in 28 cevarum hybrids. Albertin et al. (2013) detected recombinant mtDNA in 10% of their cevarum hybrids. Verspohl et al. (2018) found recombinant mitotypes in three out of 83 hybrids. The different results can be attributed to different strains used for hybridisation or to different detection methods. Because of the lower resolution genotyping approaches in the previous studies many recombination events might have gone undetected. Verspohl et al. (2018) included only three genes (*COX2*, *COX3*, and *ATP6*) in the analysis. When we consider only those genes, 5 of our hybrids can be categorized as having *S. uvarum* mitochondrial genomes. In our analysis we combined whole-genome RFLP with the comparison of the amplified segments of four protein-encoding genes. In *S. cerevisiae*, mtDNA encodes eight proteins, such as subunits of the respiratory complexes III (Cytb), IV (Cox1, Cox2, and Cox3) and V (Atp6, Atp8, and Atp9) (Rps3/Var1) (Foury et al., 1998). We used 4 of these genes for the detection of recombination. With the combined approach we found mitotypes different from both parental mitotypes at a frequency (34%) comparable to the recombination frequencies found in *S. cerevisiae* intraspecies crosses. Dujon et al. (1974) described a recombination rate in crosses of *S. cerevisiae* strains of up to 25%. Recently Wolters et al. (2018) observed that approximately 40% of the mated *S. cerevisiae* cells contained recombinant mtDNAs.

The astonishingly high proportion of cevarum hybrids with recombinant mitotypes implies that the *S. cerevisiae* mitochondria can fuse easily with the *S. uvarum* mitochondria during conjugation to let their DNAs physically interact and then the fused mitochondria divide (fission) to allow the separation of the recombinant (chimeric) genomes from the parental genomes. It has been shown that in vegetatively propagating cells, the mitochondria undergo constant fusion and fission events (Nunnari et al., 1997; Westermann, 2010; Murley et al., 2013; Higuchi-Sanabria et al., 2016). Analogous processes may take place in the cevarum zygotes.

Given that the mitochondrial genomes of the two species differ in size and gene order (e.g., Cardazzo et al., 1998; Ruan et al., 2017), the observed rate is surprisingly high. The mtDNA of the *S. uvarum* type strain is smaller by nearly one third than the mitochondrial genome of the *S. cerevisiae* neotype strain (Sulo et al., 2017) and certain segments have different and even inverted positions in the two species (Ruan et al., 2017; Sulo et al., 2017). It can be assumed that because of the synteny differences, multiple recombination events are required for the formation of complete chimeric marker sets (co-segregation of markers inherited from different parents). Interestingly, recombination took place mainly outside of the markers, which might be attributed to the preferential location of recombination hotspots in non-protein-coding regions of the genomes. In *S. cerevisiae*, recombination occurs mainly in the intergenic GC-rich regions (e.g., Dieckmann and Gandy, 1987; Fritsch et al., 2014). Another interesting feature of the recombinant mitochondrial genomes is the high representation of the *S. uvarum* alleles of *COX2* and *COX3*. Their high occurrence implies that either recombination does not occur randomly between the mitochondrial genomes of the partner species or the observed allele (ortholog) combinations fit the alloploid nuclear genome better than other combinations.

### The Sterility of the Hybrids and Its Break-Down Are Not Affected by Mitotype

Viable spores were produced in all but one hybrid regardless of their mitotypes. Thus, the cevarum nuclear genome can accommodate either of the parental mitochondrial genomes and also their recombinant versions with no severe impact on meiosis and sporulation. Nonetheless, the viability of the spores of these hybrids does not contradict the observations that certain nuclear genes of one species are incompatible with the mitochondria of the other species and their incompatibility causes sporulation deficiency (Lee et al., 2008; Chou et al., 2010; Špírek et al., 2015; Jhuang et al., 2017). Those incompatibilities were detected in segregants of sporulation-proficient hybrids and were usually recessive. In the alloploid nuclei of the cevarum hybrids the dominant compatible alleles are also present for both the *S. cerevisiae* and the *S. uvarum* mitochondria. The nucleo-mitochondrial conflict therefore cannot play a role in hybrid sterility.

Due to the second sterility barrier, the overwhelming majority of the spores were sterile allodiploids but one third of the hybrids produced also at least a few aneuploid spores that lost the *MAT* heterozygosity responsible for the sterility of the allodiploid spores. The break-down of the sterility barrier did not correlate with any mitotype; leu^–^ segregants nullisomic for the *MAT*-carrying chromosome of *S. uvarum* were produced independently of the mitotypes of the hybrids. This finding indicates that the restoration of fertility by malsegregation in tetraploid meiosis is not affected by the mitotype.

### Impaired Thermotolerance of Respiratory Growth in Hybrids With Recombinant Mitochondrial Genomes

By (taxonomic) definition, *S. cerevisiae* tolerates high temperatures, whereas *S. uvarum* (formerly also known as *S. bayanus* var. *uvarum*) is inhibited by temperatures above 34°C (Vaughan-Martini and Martini, 1993) but numerous studies described strains of the latter species that could grow also at high temperatures (for a review, see Sipiczki, 2002 and references therein). All hybrids generated in this study grew well on glucose-containing medium at 37°C regardless of their mitotypes. Thus, the mitotype is not involved in the determination of temperature tolerance (of the hybrid) or it may play only a minor role. This finding is consistent with the observation that cevarum hybrids with no functional mitochondria can grow at this temperature (Li et al., 2019) and with the findings suggesting that the main genes determining temperature tolerance are not in the mitochondrial but in the nuclear genome (Serra et al., 2005).

However, this conclusion does not hold true for media containing glycerol as the energy and carbon source. In a recent study, cevarum hybrids with *S. uvarum* mitotype exhibited reduced growth intensity on a glucose medium and almost no growth on the glycerol medium at 37°C (Li et al., 2019). Unexpectedly, our experiments could not confirm this correlation. Our hybrids showing *S. uvarum*-type RFLP grew well on both media at 37°C. In contrast, the hybrids that did not grow on glycerol at 37°C had recombinant mitochondria lacking the *S. cerevisiae* orthologs of the *ATP6*, *COX3*, and *COX2* genes. But they grew on glycerol at 25°C, indicating that they were not petite. This correlation is consistent with the temperature sensitive glycerol utilization (respiratory growth) recently observed upon the replacement of the *S. cerevisiae* genes *COX1* and *ATP6* with their *S. uvarum* counterparts in the *S. cerevisiae* mitochondrial genome (Li et al., 2019). The replacement of *COX2* and *COX3* only reduced the efficiency. Interestingly, the only non-sporulating hybrid found in our study was defective in glycerol utilization at 25°C as well. It had recombinant mtDNA. Albertin et al. (2013) found two hybrids with recombinant mitotypes in a collection of 20 cevarum hybrids. One of them was unable to respire. It is pertinent to note here that a recent study demonstrated that certain allele combinations of mitochondrial genes can impair the growth on glycerol at high temperature even in S. *cerevisiae* intraspecies hybrids (Wolters et al., 2018).

### Meiotic Instability of Recombinant Mitotypes

Because of the autodiploidisation of their meiosis, the allotetraploid cells produce viable but sterile allodiploid F1 spores. As the process is error-prone, fertile alloaneuploid spores are occasionally also formed (see above). The alloaneuploid spore monosomic for the *MAT*-carrying chromosome produces fertile vegetative cells that are prone to lose additional chromosomes from one or the other subgenome during subsequent meiotic divisions (GARMe) (Karanyicz et al., 2017). If the recurrent loss of chromosomes is accompanied with recombination between allosyndetic (homeologous) chromosomes, GARMe results in chimeric nuclear genomes. In this study we find that the mitochondrial genomes of the cevarum hybrids can also undergo changes during meiosis. The F1 spore clones produced by hybrids of recombinant (chimeric) mitochondrial genomes frequently had whole-genome RFLP patterns different from those of the hybrids. Assuming homoplasmy in these hybrids, we inferred that the pattern changes could be attributed to intramolecular rearrangements and ectopic recombination. The structural alterations of the mtDNA structure were associated with the loss of the ability to utilize glycerol as the only carbon source (petite phenotype) and sporulation deficiency in almost all mitochondrial segregants. Thus, the recombination of the parental mitochondrial genomes during hybridisation generates mtDNA molecules with inherent instability associated with potential fatal consequences on mitochondrial functions. Cevarum hybrids producing F1 spore clones unable to grow on glycerol were previously described by Sebastiani et al. (2002), but the background of the segregation was not investigated.

### Evolutionary Considerations

The postzygotic reproductive isolation of the *Saccharomyces* species seems to be the result of the combined and synergistic effects of several factors, such as (1) the sequence and structural differences between the homeologous (allosyndetic) chromosomes (impairing chromosome pairing in the prophase of meiosis-I), (2) mating-type heterozygosity (repressing the mating process) and (3) functional incompatibility of certain nuclear and mitochondrial genes (preventing sporulation in certain segregant spore clones by causing respiration deficiency). None of these mechanisms appears sufficient to ensure total reproductive isolation of the species because each can be overcome: (1) genome duplication overrides the chromosomal pairing problem (by allowing autodiploidisation of the allopolyploid meiosis; Karanyicz et al., 2017), (2) loss of *MAT* heterozygosity (by malsegregation of *MAT*-carrying chromosomes; Pfliegler et al., 2012) abrogates the suppression of conjugation, and (3) rearrangements (chimerisation) in the hybrid nuclear genome accompanied with recombination of the parental mitochondrial genomes creates segregants avoid of incompatible combinations of nuclear and mitochondrial genes (Lee et al., 2008; Chou et al., 2010) and results of this study.

**FIGURE 7:**
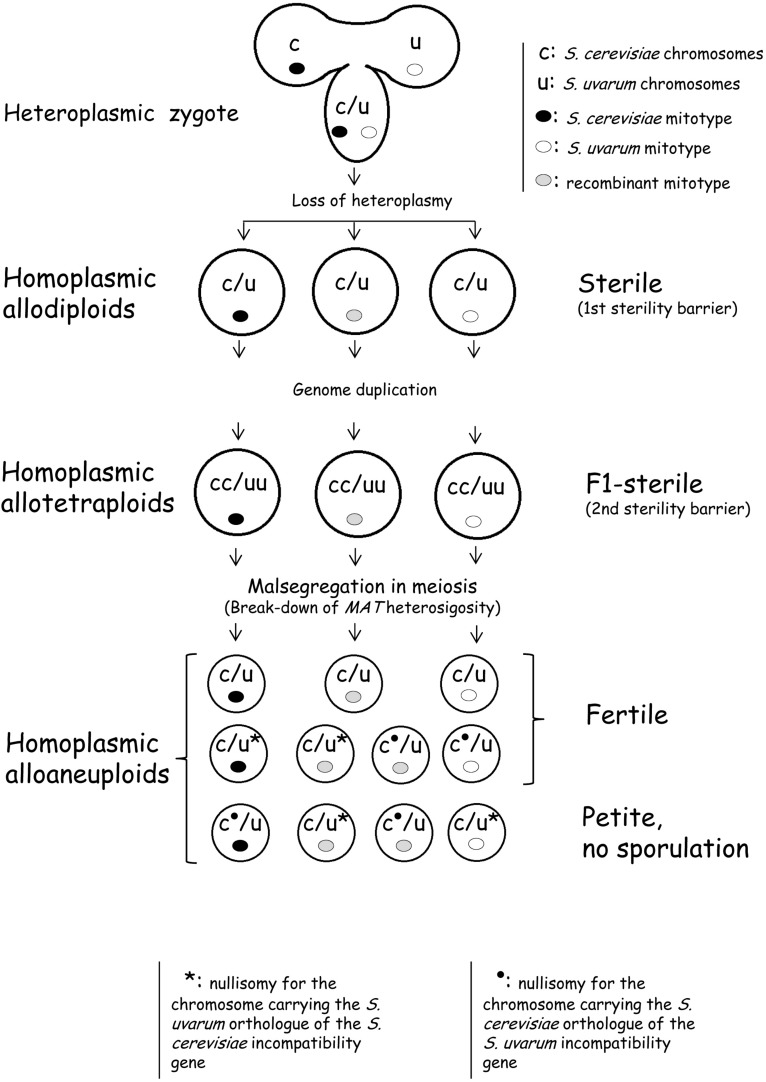
A model depicting the postzygotic evolution of the nuclear and mitochondrial genomes of sterile cevarum hybrids in which the sterility barrier breaks down and both fertile and sterile segregants are formed.

When concerning the involvement of the mitochondrial genomes in reproductive isolation of species, one has to bear in mind that the nucleo-cytoplasmic incompatibilities do not cause sporulation deficiency directly in the hybrids but only in their segregants (e.g., spore clones) that lose the heterozygosity of the nuclear genes by inheriting only the alleles (orthologs) incompatible with the mitochondrion (Lee et al., 2008; Chou et al., 2010). As only a few incompatible combinations are known (Lee et al., 2008; Chou et al., 2010; Špírek et al., 2015; Jhuang et al., 2017), considerable proportions of the spores can be fertile. These represent escape routes from the sterility caused by nucleo-mitochondrial incompatibility. Besides, even the spores inheriting incompatible gene combinations are only partially sterile, because respiration-deficiency, which prevents sporulation, does not represses mating (Clark-Walker and Miklos, 1975). It has also to be taken into consideration that incompatible alleles occur also in conspecific strains (e.g., Codón et al., 1995) and some interspecies incompatibilities appear to be strain-specific only (Sulo et al., 2003). Figure 7 shows a model of the postzygotic evolution of the nuclear and mitochondrial genomes of cevarum hybrids producing both sterile and fertile segregants due to the break-down of the second sterility barrier. The replacement of mitochondrial genes in certain *Saccharomyces* wine strains with orthologs from a different species, promiscuously called horizontal gene transfer (HTG) and/or introgression (without any clues on the transfer mechanism) (Peris et al., 2017) can be attributed to processes similar to those depicted in Figure 7. If nucleo-mitochondrial incompatibility made all spores sterile, it would block gene flow between populations of different species (as hypothesized by Chou et al., 2010), but then no *Saccharomyces* strains with chimeric genomes could have been produced in the nature and no fertile filial spore clones described in studies on synthetic hybrids could be obtained after the loss of *MAT* heterozygosity (reviewed in Sipiczki, 2018). The nucleo-mitochondrial incompatibility seems to play a minor indirect role in the biological isolation of the *Saccharomyces* species by rendering the cells of certain spore clones (but not the hybrid cells) deficient in respiration. The non-respiring cells propagate more slowly than the respiring cells.

Neither postzygotic reproductive isolation nor mitonuclear incompatibilities are specific to *Saccharomyces*; both phenomena are known in other groups of fungi as well (e.g., Olson and Stenlid, 2001; Le Gac et al., 2007; De Vienne et al., 2009; Turner et al., 2011; Giordano et al., 2018; Verma et al., 2018) and the *MAT* loci were also implicated in the isolation of other species (e.g., Ortiz-Merino et al., 2017; Watanabe et al., 2017; Braun-Galleani et al., 2018; Bizzarri et al., 2019). However, the current knowledge on the role and the effect of mitonuclear interactions if fungal hybrids is still limited and mainly focused on virulence (e.g., Olson and Stenlid, 2001; Verma et al., 2018; Giordano et al., 2018).

## Conclusion

The results indicate that in the petite-positive genus *Saccharomyces*, in contrast to metazoans, the nucleo-mitochondrial incompatibility is only marginally involved in the reproductive barrier. The nuclear genome of the cevarum (*S. cerevisiae* × *S. uvarum*) hybrid is compatible with both parental mitochondrial genomes and their recombinants. Our previous studies (e.g., Pfliegler et al., 2012) demonstrated that the allopolyploid (allotetraploid) cells produce viable, mostly sterile alloploid (allodiploid) ascospores, but the loss (malsegregation) of the *MAT*-carrying chromosome of one of the subgenomes during meiosis (loss of *MAT*-heterozygosity) results in (alloaneuploid) ascospores that can act as gametes because *MAT* homozygosity reactivates the mating (fertilization) process. The results of this study show that neither the sterility nor the fertility of the ascospores (gametes) can be associated with parental or recombinant mitochondrial genomes. Nucleo-mitochodrial incompatibility was observed only in certain recombinant ascospores as respiration deficiency. As respiration is not essential for life in *Saccharomyces*, these gametes produce vegetative clones of cells which can fertilize (conjugate with) each other or the cells of other spore clones of different mating type. From these results we concluded that the sterility barrier separating the *Saccharomyces* species is due to the failure of allosyndetic (homeologous) chromosomes to pair and to mating-type (*MAT*) heterozygosity (described in detail in our previous works and reviewed in Sipiczki, 2018), rather than to nucleo-mitochondrial incompatibilities. The postzygotic evolution of the mitochondrial genomes including interspecies recombination may have evolutionary relevance and the analysis of an even larger number of hybrids could contribute to the better understanding of the evolutionary processes that have shaped the yeast mitochondrial genomes (Freel et al., 2015; Wolters et al., 2018).

## Data Availability Statement

All datasets generated for this study are included in the article/Supplementary Material.

## Author Contributions

MS and ZA designed the experiments. AS and EK performed the experiments. MS provided funding for this research, wrote the manuscript, and performed the data analysis. AS, EK, and ZA participated in manuscript revision.

## Conflict of Interest

The authors declare that the research was conducted in the absence of any commercial or financial relationships that could be construed as a potential conflict of interest.

## Footnotes

1 https://www.ebi.ac.uk/Tools/psa/emboss_matcher/
