# Supplementary material for: Diversity and Postzygotic Evolution of the Mitochondrial Genome in Hybrids of Saccharomyces Species Isolated by Double Sterility Barrier
Source: Front Microbiol. 2020 May 7;11:838. doi: 10.3389/fmicb.2020.00838 (PMC7221252; doi:10.3389/fmicb.2020.00838)
Supplement: Supplementary file 1 [file Presentation_1.PPTX]

## Slide 1
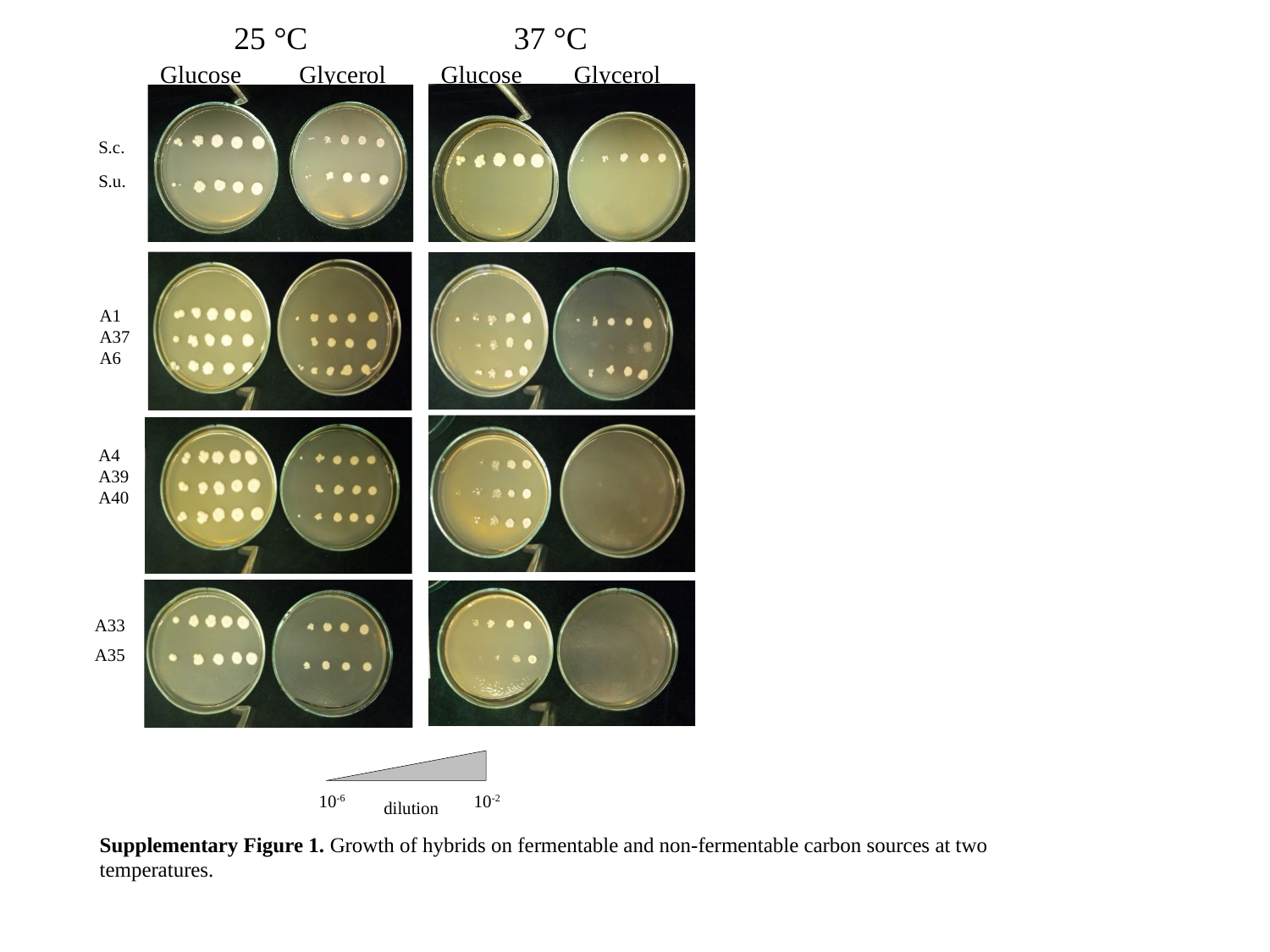

25 °C
37 °C
Glycerol
Glucose
Glucose
Glycerol
S.c.
S.u.
A1
A37
A6
A4
A39
A40
A33
A35
10-6 10-2
dilution
Supplementary Figure 1. Growth of hybrids on fermentable and non-fermentable carbon sources at two temperatures.
